# P-550. Efficacy and Safety of B/F/TAF in Black Adults with HIV Who Are Treatment Naïve: 5-Year Follow-Up from Two Phase 3 Studies

**DOI:** 10.1093/ofid/ofae631.749

**Published:** 2025-01-29

**Authors:** Anson K Wurapa, Jose Ramon Arribas, Yazdan Yazdanpanah, Chloe Orkin, Hui Liu, Rachel Rogers, David Malebranche, Jason Hindman, Debbie P Hagins

**Affiliations:** Infectious Disease Specialists of Atlanta, Atlanta, Georgia; Hospital Universitario La Paz, Madrid, Madrid, Spain; Bichat–Claude Bernard Hospital, Paris, Ile-de-France, France; Queen Mary University of London, london, England, United Kingdom; Gilead Sciences, Inc., Foster City, California; Gilead Sciences, Inc., Foster City, California; Gilead Sciences, Foster City, California; Georgia Department of Public Health, Coastal Health District, Chatham CARE Center, Savannah, GA, USA, Savannah, Georgia

## Abstract

**Background:**

Black communities are disproportionately affected by HIV and may have a greater lifetime risk of comorbidities compared with non-Black people with HIV (PWH). Despite this, they have historically been underrepresented in clinical studies. Here, we report efficacy and safety through 5 years of first-line therapy with bictegravir/emtricitabine/tenofovir alafenamide (B/F/TAF) in Black PWH.

**Figure:**
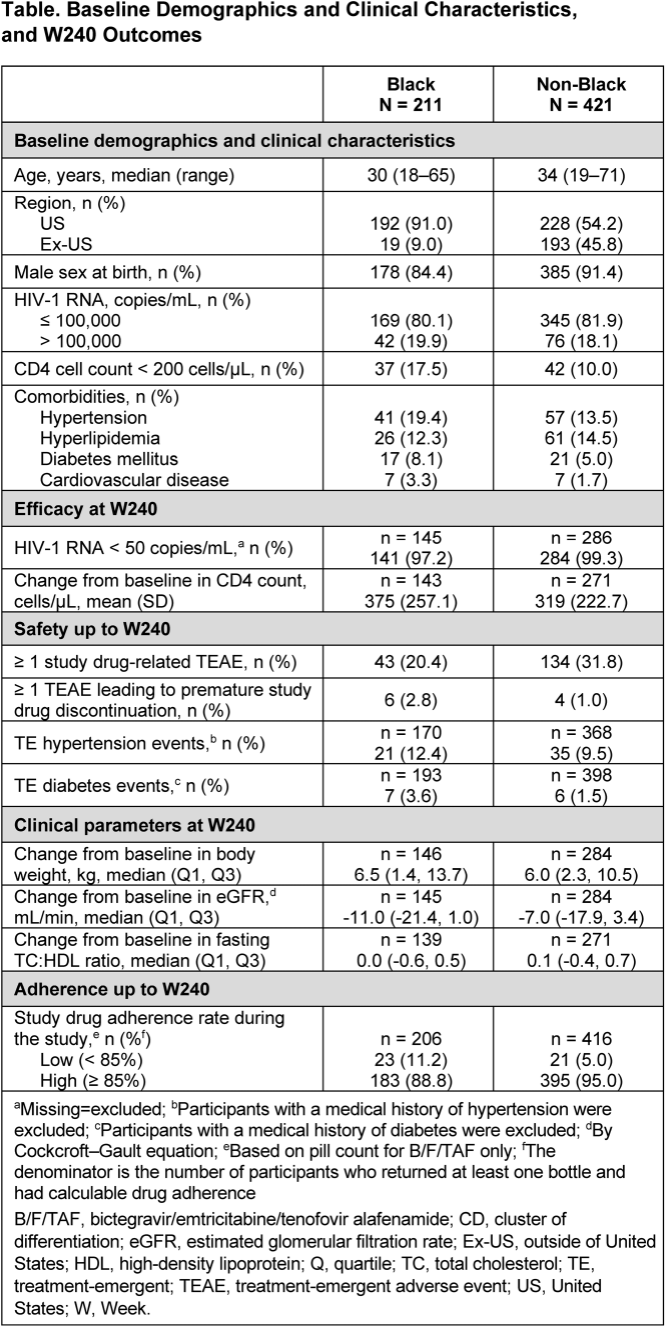

**Methods:**

Studies 1489 (NCT02607930; B/F/TAF vs dolutegravir/abacavir/lamivudine [DTG/ABC/3TC]) and 1490 (NCT02607956; B/F/TAF vs DTG+F/TAF) were randomized, double-blind, multicenter, Phase 3 studies in adult PWH. This pooled analysis reports the outcomes of Black and non-Black participants who received B/F/TAF in the 144-week (W) randomization phase and the 96W open-label extension. Baseline demographics and clinical characteristics; proportion of participants with HIV-1 RNA < 50 copies/mL (missing=excluded); adherence; treatment-emergent adverse events (TEAEs); and changes in CD4 count and metabolic and renal parameters are presented.

**Results:**

Overall, 211 (33.4%) Black and 421 (66.6%) non-Black PWH (91.0% and 54.2% from the US, respectively) received B/F/TAF up to W240. Baseline demographics and clinical characteristics are shown in the Table; 19.9% Black and 18.1% non-Black PWH had HIV-1 RNA > 100,000 copies/mL, 17.5% and 10.0% had CD4 < 200 cells/µL, and 19.4%/8.1% and 13.5%/5.0% had a history of hypertension/diabetes mellitus, respectively. At W240, high proportions of both Black (97.2%) and non-Black PWH (99.3%) had HIV-1 RNA < 50 copies/mL, despite Black PWH being more likely than non-Black PWH to have low (< 85%) adherence (11.2% vs 5.0%; *P*=0.0074 [Fisher’s exact test]). A smaller proportion of Black vs non-Black PWH experienced study drug–related TEAEs (20.4% vs 31.8%; *P*=0.0026 [Fisher’s exact test]). Changes in CD4 count, metabolic and renal parameters, and TE hypertension and diabetes were similar between groups (Table).

**Conclusion:**

At Year 5, B/F/TAF maintained high rates of virologic suppression in Black PWH, despite a greater proportion of Black vs non-Black PWH having low adherence. B/F/TAF was well tolerated, with a smaller proportion of Black vs non-Black PWH with study drug–related TEAEs. These findings further support the long-term use of B/F/TAF in Black PWH.

**Disclosures:**

**Anson K. Wurapa, MD**, Gilead: Honoraria|Gilead Sciences, Inc.: Medical writing support provided by Aspire Scientific (Bollington, UK) **Jose Ramon Arribas, MD**, Alexa: Advisor/Consultant|Gilead Sciences, Inc.: Advisor/Consultant|Gilead Sciences, Inc.: Expert Testimony|Gilead Sciences, Inc.: Grant/Research Support|Gilead Sciences, Inc.: Medical writing support provided by Aspire Scientific (Bollington, UK)|Janssen: Advisor/Consultant|Janssen: Expert Testimony|Lilly: Advisor/Consultant|MSD: Advisor/Consultant|MSD: Expert Testimony|Serono: Advisor/Consultant|Teva: Advisor/Consultant|ViiV: Advisor/Consultant|ViiV: Expert Testimony|ViiV: Grant/Research Support **Yazdan Yazdanpanah, MD, PhD**, Gilead Sciences, Inc.: Medical writing support provided by Aspire Scientific (Bollington, UK) **Chloe Orkin, MBChB, FRCP, MD**, AstraZeneca: Grant/Research Support|Gilead Sciences, Inc.: Grant/Research Support|Gilead Sciences, Inc.: Honoraria|Gilead Sciences, Inc.: Medical writing support provided by Aspire Scientific (Bollington, UK)|GSK: Honoraria|MSD: Honoraria|ViiV Healthcare: Grant/Research Support|ViiV Healthcare: Honoraria **Hui Liu, PhD**, Gilead Sciences, Inc.: Employee; Medical writing support provided by Aspire Scientific (Bollington, UK)|Gilead Sciences, Inc.: Stocks/Bonds (Private Company) **Rachel Rogers**, Gilead Sciences, Inc.: Employee; Medical writing support provided by Aspire Scientific (Bollington, UK)|Gilead Sciences, Inc.: Stocks/Bonds (Private Company) **David Malebranche, MD, MPH**, Gilead Sciences, Inc.: Employee; Medical writing support provided by Aspire Scientific (Bollington, UK)|Gilead Sciences, Inc.: Stocks/Bonds (Private Company) **Jason Hindman, PharmD, MBA**, Gilead Sciences, Inc.: Employee; Medical writing support provided by Aspire Scientific (Bollington, UK)|Gilead Sciences, Inc.: Stocks/Bonds (Private Company) **Debbie P. Hagins, MD, FAPCR, AAHIVS**, Gilead Sciences, Inc.: Medical writing support provided by Aspire Scientific (Bollington, UK)

